# Probable Mechanism(s) of Antifungal Activity of SJA-95, a Heptaene Polyene Antibiotic

**DOI:** 10.4103/0250-474X.41449

**Published:** 2008

**Authors:** S. K. Desai, S. R. Naik

**Affiliations:** Department of Pharmacology and Biotechnology, Prin. K. M. Kundnani College of Pharmacy, Plot No 23, Jote Joy Building, R. S. Marg, Cuffe Parade, Mumbai-400 005, India

**Keywords:** Antifungal, cholesterol, ergosterol, heptaene, preferential binding

## Abstract

A new strain, streptomyces sp. S. 24 was isolated from a soil sample collected from Japan. The strain produced heptaene polyene antibiotic, SJA-95, in submerged culture and found to elicit promising antifungal activity against yeasts, filamentous fungi and clinical isolates, both in vitro and in vivo. Experimental studies were carried out using biological methods to understand the probable mechanism(s) of antifungal activity of SJA-95. Our experimental findings suggest that SJA-95 binds more avidly to ergosterol, the sterol in fungal cell membranes, than to cholesterol found in mammalian cell membranes. Such preferential binding of SJA-95 to ergosterol might help to establish its usefulness as a chemotherapeutic agent with lesser adverse reactions.

During a soil screening program for antifungal antibiotics, a new strain S.24 was isolated from a soil sample collected from Japan. The strain produced heptaene polyene antibiotic (SJA-95) in submerged culture and found to elicit promising antifungal activity against yeasts, filamentous fungi and clinical isolates including plant pathogens, both *in vitro* and *in vivo*^1^. Documented research findings suggest antifungal activity of polyenes largely attributable to their binding to cholesterol and ergosterol, the two principal sterols found in eukaryotic cell membranes^2^,^3^. If a polyene antibiotic is to kill the fungal organism, it must selectively bind to ergosterol which causes distortion and structural rearrangement so that the membrane is destabilized resulting in abnormal function viz: Efflux of cellular K^+^ ions^4^,^5^. Such preferential binding of polyene antibiotics to ergosterol would thus help determine its clinical usefulness by reducing toxicity to normal mammalian cells. The present investigation attempts to find out the nature and specificity of the binding of SJA-95 to sterols of fungal and mammalian cell membranes with an objective to understand the possible underlying mechanism (s) of action.

## MATERIALS AND METHODS

SJA-95 was obtained as a gift sample from Hindustan Antibiotics, Pune. Cholesterol and ergosterol were obtained from Sigma Chemicals (St. Louis, Mo). All other chemicals and reagents were of AR Grade and obtained from local suppliers. SJA-95 stock solution was prepared in dimethyl sulfoxide (DMSO). Sterols were dissolved in 2-propanol and used within 7 days. *Candida albicans* (ATCC no 10231) culture was obtained from National Collection of Industrial Microorganisms (NCIM), National Chemical laboratory, Pune-411 008 and maintained on Sabouraud’s agar medium. Erythrocytes were obtained from anticoagulated blood of healthy human volunteers. The experimental protocol to study the binding affinity of SJA-95 to ergosterol and cholesterol was designed as per the method described by Nadeau *et al*,^6^ with minor modifications.

### Effect of addition of various chemicals on the MIC of SJA-95:

The influence of addition of various chemicals like sorbitol, bovine albumin, cholesterol and ergosterol to the assay medium (Sabouraud’s broth) was studied by determining minimum inhibitory concentration (MIC) values of SJA-95 against *Candida albicans*. Colonies of *Candida albicans* grown on Sabouraud’s agar medium for 48 h were dispersed in sterile broth and compared with McFarland standard to give a starting inoculum of 1×10^6^-5×10^6^ organisms/ml. The broth medium was further diluted to obtain a cell suspension ranging from 1×10^4^-5×10^4^ organisms/ml and used for determining MIC of SJA-95. A tenfold concentrated drug solution (0.1 ml) was added aseptically to 0.9 ml of the broth medium containing sorbitol (0.8, 3.2 mM), bovine albumin (1%), cholesterol (1, 3 μg/ml) and ergosterol (1, 3 μg/ml) respectively and incubated at 32° for 48 h. Protection of yeast cells from the action of SJA-95 was assessed by the change in MIC values. Controls using the chemicals without the drug as also solvent controls were run simultaneously.

### Binding affinity of SJA-95 to sterols using human erythrocytes:

Erythrocytes were separated by centrifugation from anticoagulated blood of healthy donors, washed twice with phosphate buffer saline (PBS pH-7.4) and adequately diluted with PBS to a strength of 10^8^ cells/ml by counting on a haemocytometer. The cell suspension was stored in a refrigerator and used within 24 h of preparation. Different concentrations of sterols in 2-propanol were added to a series of test tubes containing 5 ml PBS, SJA-95 solution in DMSO (10 μl) was then added to the tubes and incubated at 37° for 30 min to allow for complex formation. Care was taken to ensure that the concentration of the solvents did not exceed 1%. After incubation cell suspension containing 5×10^7^ erythrocytes was added to each tube and incubated for 15 min. Positive and negative control tubes with and without SJA-95 along with erythrocytes in PBS were run simultaneously. The tubes were then centrifuged at 3000 rpm for 10 min, supernatants removed and cell pellets washed twice with PBS. The washed pellets were reconstituted with 4 ml distilled water and the haemoglobin content from the haemolysed erythrocytes determined by measuring its absorbance at 520 nm on spectrophotometer (Jasco, V-550 UV/Vis Spectrophotometer).

### Binding affinity of SJA-95 to sterols using *Candida* cells:

*Candida albicans* cells were grown in Sabouraud liquid medium at 32° for 36 h, cells separated by centrifugation at 3000 rpm for 10 min, rinsed twice with PBS, dispersed in PBS and counted on a haemocytometer. The conc. of the cells was adjusted approximately to 10^7^ cells/ml. Different concentrations of sterols in 2-propanol were added to a series of test tubes containing 5 ml PBS, SJA-95 in DMSO (10 μl) was added to the tubes and after thorough mixing incubated at 32° for 30 min as described earlier. After incubation, 2×10^7^ cells in 2 ml PBS were added to each tube and further incubated at 32° for 1 h with intermittent agitation on a vortex mixer. The cells were then separated by centrifugation, supernatants removed and cell pellets washed with PBS. The washed pellets were then dispersed in 15 mM lithium carbonate solution, boiled in a waterbath for 5 min and centrifuged at 3000 rpm for 10 min. The K^+^ concentration in the supernatant was measured with a flame photometer (Digital Flame Photometer, Unit 121, Systronics Fiebig).

In another set of experiments, tubes containing 5 ml of PBS containing cell suspension as described earlier, were incubated with the drug solution (10 μg) at 32° to which 85 mM KCl (1 ml) and 45 mM MgCl_2_ (1 ml) respectively were added before and 24 h after incubation. Growth was checked at the end of 48 h.

### Sterol interaction with SJA-95 by spectrophoto-metric method:

Stock solution of SJA-95 (1 mg/ml) was prepared in DMSO and further diluted with distilled water to get a concentration of 20 μg/ml. The binding/interaction of SJA-95 with cholesterol and ergosterol was studied by mixing the drug solution with the respective sterol solution (20 μg/ml) in equal proportion followed by incubation at 32° for 60 min. The spectra of the complex formed (SJA-95 and cholesterol or SJA-95 and ergosterol) were recorded immediately after incubation on Jasco spectrophotometer. Thereafter equal volume of isopropanol was added to the mixture complex formed and spectra were recorded.

## RESULTS AND DISCUSSION

Sorbitol is a common osmotic protectant used in stabilizing fungal protoplasts. In sorbitol protection and morphology assay, sorbitol did not inhibit/block the effect of SJA-95 at both the doses studied and there was no shift in MIC values (Table 1). The morphology of *Candida* cells grown in presence of sorbitol on the 7^th^ day showed normal cells in small clusters of 4-5 cells with intermediate budding cells, typical of yeasts. Bovine albumin (1%) also failed to change the MIC value of SJA-95. In another set of experiments, a dose related shift in MIC of SJA-95 against *Candida albicans* was seen in presence of sterols. Ergosterol (3 μg/ml) showed maximum shift in MIC of SJA-95 from 0.5 μg/ml to 6 μg/ml (Table 1). The relative affinity of SJA-95 to ergosterol seems to be higher than cholesterol which might have resulted in greater protection to yeast cells.

**TABLE 1:** EFFECT OF ADDITION OF VARIOUS CHEMICALS ON THE MIC VALUES OF SJA-95 AGAINST *CANDIDA ALBICANS*

Description	MIC (μg/ml)
SJA-95 (Control)	0.5
SJA-95 + Sorbitol 0.8 mM	0.5
SJA-95 + Sorbitol 3.2 mM	0.5
SJA-95 + Bovine albumin 1%	0.5
SJA-95 + Cholesterol 1 μg/ml	1
SJA-95 + Cholesterol 3 μg/ml	2
SJA-95 + Ergosterol 1 μg/ml	2
SJA-95 + Ergosterol 3 μg/ml	6

Sterols, particularly with 27 to 29 C atoms (e.g. ergosterol, cholesterol) are major constituents of fungal cell membrane and it has been experimentally demonstrated that sterols antagonize the action of polyene antibiotics and the latter is shown to interfere with the sterol biosynthesis of fungal membrane^7^,^8^. Furthermore it has been reported that exogenously added sterols facilitate the fungi to overcome the blockade of biosynthesis by polyene antibiotics^9^–^11^. Our experimental findings have shown the sterols protecting the erythrocytes from haemolysis in a dose dependent manner (Table 2). Ergosterol showed greater protection as evidenced by the increased retention of haemoglobin. Considering the haemolysis produced by control as 100%, maximum protection was observed with ergosterol at drug sterol concentration ratio of 0.1 (Table 2). Furthermore ergosterol treatment also showed greater protection of *candida* cells as reflected by increased retention of K^+^ ions largely by blocking the action of SJA-95 (Table 3).

**TABLE 2:** CHOLESTEROL AND ERGOSTEROL PROTECTION TO RBCS

Drug:Sterol (conc. ratio)	% Protection (Cholesterol)	% Protection (Ergosterol)
Control	0	0
10	8.32+3.66	9.05+3.10
5	15.81+5.81	20.11+1.90
2	23.36+7.37	37.17+5.41*
1	32.48+7.14*	52.53+6.28*
0.5	41.87+4.32*	55.02+7.34*
0.2	57.25+6.5*	72.86+7.56*
0.1	65.29+5.78*	88.45+6.09*

Each value represents mean of 4 readings ±SEM. Treatment groups compared with control (% inhibition of cell lysis). One-way ANOVA using Dunnett’s test is applied for statistical analysis.

* significant at *p* <0.01

**TABLE 3:** CHOLESTEROL AND ERGOSTEROL PROTECTION TO *CANDIDA ALBICANS*

Drug: Sterol (conc. Ratio)	% Protection (Cholesterol)	% Protection (Ergosterol)
Control	0	0
10	18.44±2.44	25.98±3.09*
5	44.34±9.05*	72.63±4.95*
2	64.93±6.65*	101.1±5.35*
1	78.48±6.34*	104.85±6.7*
0.5	81.53±1.96*	120.23±2.58*
0.2	92.4±5.67*	123.35±2.16*
0.1	96.62±2.48*	125.1±1.04*

Each value represents mean of 4 readings ±SEM. Treatment groups compared with control (% inhibition of cell lysis). One-way ANOVA using Dunnett’s test is applied for statistical analysis.

* significant at *P* <0.01

**Fig 1:**
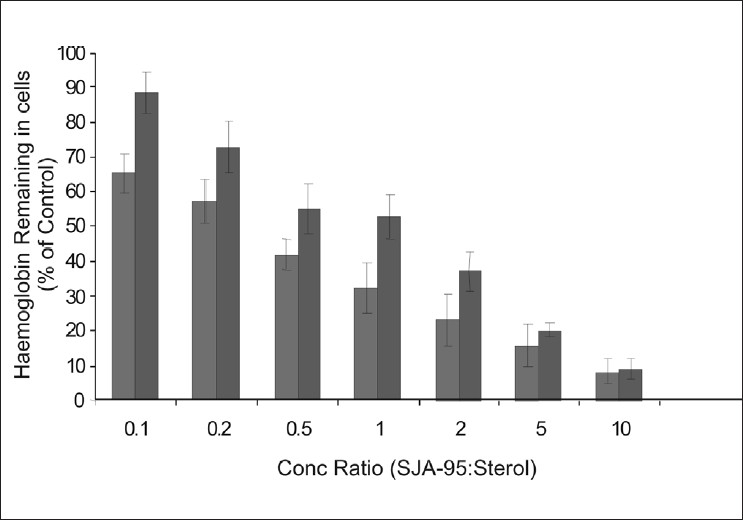
Effects of Cholesterol and Ergosterol on SJA-95 induced lysis of erythrocytes. Comparison of exogenously added cholesterol (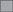) and ergosterol (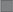) protection to erythrocytes at SJA-95:Sterol concentration ratios ranging from 0.1 to 10.

**Fig. 2:**
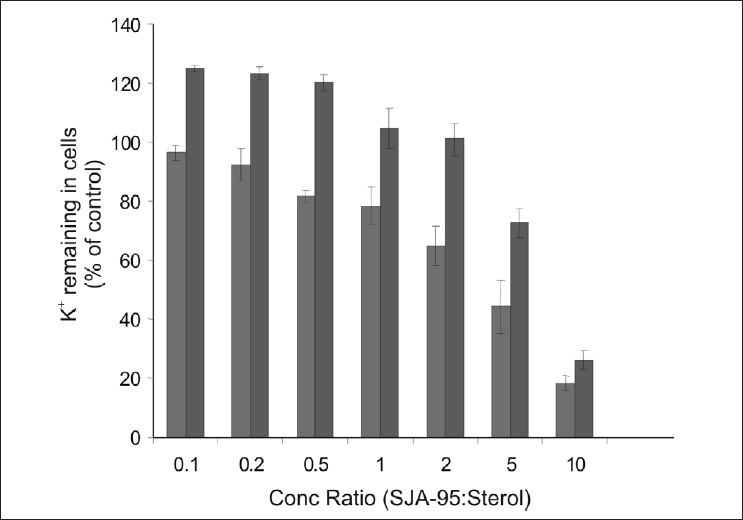
Effects of Cholesterol and Ergosterol on SJA-95 induced K+ leakage from *Candida albicans*. Comparison of exogenously added cholesterol (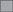) and ergosterol (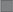) protection to Candida cells at SJA-95:Sterol concentration ratios ranging from 0.1 to 10.

A comparative depiction of the sterols^’^ protection to RBCs and *Candida* cells highlights the preferential binding and greater protection offered by ergosterol both to the erythrocytes as well as fungal cells (figs. 1 and 2). An interesting observation showed K^+^ retained in cells reaching 100% of control values at drug sterol concentration ratio of 2 and further rising to 120% of control values (fig. 2). As the K^+^ values at different sterol concentrations were calculated considering lysis occurring in control groups as 100%, these observations were found intriguing. At higher concentrations, ergosterol was not only able to reverse the biological activity of the polyene but it probably further protected the cell membrane and prevented leakage of vital constituents that might have occurred during the course of experiment especially during vortexing, centrifugation, washing of cell pellets etc, thus achieving K^+^ levels higher than control.

With such preferential action the degree of activity of SJA-95 will be greater on fungal cell membrane rather than mammalian cell membrane which would help to reduce the toxicity on mammalian cell membrane.

Protection of *Candida albicans* cells from the inhibitory action of SJA-95 was observed in the presence of excess K^+^ ions (85 mM KCl) as evidenced by growth of the cells after 48 h of incubation. MgCl_2_ (45 mM), however, failed to protect the cells from the action of SJA-95. Borowsky and Cybolska (1967)^12^ have also reported that inhibitory activity of N-succinylperimycin was annulled by the addition of excess K^+^ to the medium. The inhibition of glycolysis resulting in K^+^ leakage could possibly be reversed by external addition of K^+^ ions.

**Fig. 3:**
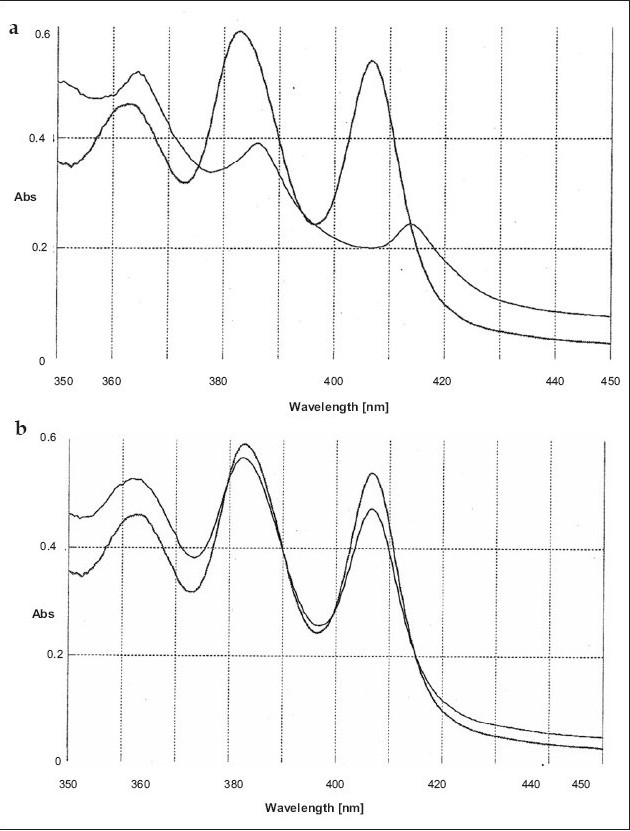
Nature of SJA-95 interaction with cholesterol. (a) Absorption spectra of SJA-95 (20 μg/ml) in phosphate buffer 7.4 after incubation at room temperature for 1 hr with (light line) and without (dark line) 20 μg/ml of cholesterol. (b) Absorption spectra of SJA-95 (20 μg/ml) in phosphate buffer 7.4 (dark line) and SJA-95 cholesterol complex after dilution with iso-propanol (1:1) (light line).

**Fig. 4:**
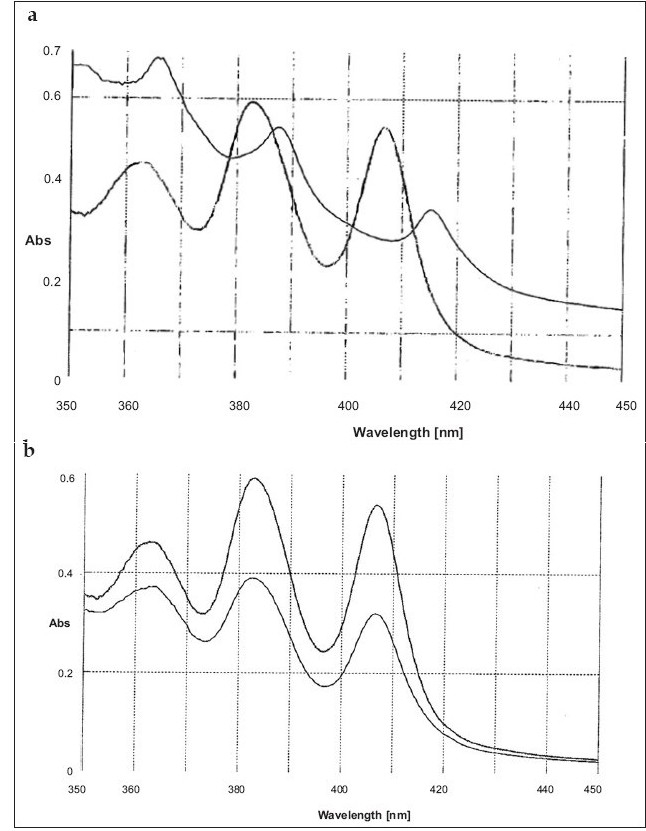
Nature of SJA-95 interaction with ergosterol. (a) Absorption spectra of SJA-95 (20 μg/ml) in phosphate buffer 7.4 after incubation at room temperature for 1 hr with (light line) and without (dark line) 20 μg/ml ergosterol. (b) Absorption spectra of SJA-95 (20 μg/ml) in phosphate buffer 7.4 (dark line) and SJA-95 and ergosterol complex after dilution with iso-propanol (1:1) (light line).

Further experiments were carried out to find out whether the nature of interaction of SJA-95 with sterols is reversible or irreversible by assessing the UV absorbance characteristics. Although both sterols produced a general lowering of absorbance peaks suggestive of a decrease in solubility of the polyene (figs. 3a and 4a), there was a slight shift in character of the spectrum in the presence of ergosterol (fig. 4a) indicating existence of interaction between the sterol and the drug. The possibility that the polyene antibiotic was destroyed in the presence of sterols could be eliminated as addition of equal volume of isopropanol to solubilize the sterol restored the absorbance of the sterol-polyene mixtures to that of the sterol free controls (figs. 3b and 4b).

On the basis of aforementioned discussion it can be concluded that SJA-95 shows preferential binding towards ergosterol of fungal cell membrane as compared to that of cholesterol of mammalian cell membrane; the binding being reversible as demonstrated by the spectral character of SJA-95. The experimentally observed mechanism(s) of action of SJA-95 might be useful in the treatment of fungal cross resistance which is devoid of adverse reactions like haemolysis. However, the direct physicochemical cell membrane damage by polyene antibiotics and ergosterol protection hypothesis needs to be further examined and established.
